# EDITORIAL COMMENT: TWO-PART SILICONE MOLD. A NEW TOOL FOR FLEXIBLE URETEROSCOPY SURGICAL TRAINING

**DOI:** 10.1590/S1677-5538.IBJU.2014.0638.1

**Published:** 2016

**Authors:** Jennifer Vlok, Homayoun Zargar

**Affiliations:** 1 Royal Melbourne Hospital, Melbourne, VIC, Australia; 2 Australian Prostate Cancer Research Centre, Melbourne, VIC, Australia

The traditional surgical training model relies on an apprentice style learning where junior doctors are gradually turned into independent surgeons through a process of observation, assisted operating, supervised operating and finally independent operating (1). All of this takes place in the operating room, with real patients and requires a senior ‘supervisor’. Limits on working hours and various other clinical and academic requirements limit operating time for junior staff. Availability of senior clinicians as teachers is also under threat as senior staff face increased patient loads and pressure by health systems to move towards consultant led care. This has led to considerable interest by surgeons in ‘model’ based learning over the past 20 years, especially as technology has progressed and minimally invasive techniques have become more numerous (2, 3). The main question is does surgical simulation transfer to skill in the operating room (3)? In urology this indeed appears to be true (4). This 2 part silicone mold presented in this video by Marroig et al. (5) appears to be a cheap, cost effective, efficient and ethical way for junior staff to familiarise themselves with flexible ureteroscopy. This model would offer a cheaper alternative to current ‘high fidelity’ ureteroscopy simulation trainers. More cost effective benchtop models have been shown to be just as useful as more expensive models (6), however the benefit of realistic model based trainers compared with cheaper computer based trainers has been questioned by some (4). The benefit of using simulation of any kind however is threefold; to the junior doctor who is able to have multiple attempts and opportunity for trial and error learning, to the hospital/senior clinician who has more efficient operating time and finally to patients whose operating time and complications may be reduced (3, 4).

*Homayoun Zargar, MD* *Department of Urology* *Royal Melbourne Hospital* *300 Grattan St, Parkville* *VIC 3050, Australia* *Email: Homi.zargar@gmail.com*

## References

[1] Roberts KE, Bell RL, Duffy AJ (2006). Evolution of surgical skills training. World J Gastroenterol.

[2] Van Sickle KR, Ritter EM, Smith CD (2006). The pretrained novice: using simulation-based training to improve learning in the operating room. Surg Innov.

[3] Sturm L (2007). Surgical Simulation for training. Skills transfer to teh operating room. ASERNIP-S Report No. 61.

[4] Preece R (2015). The current role of simulation in urological training. Cent European J Urol.

[5] Marroig B, Fortes MA, Pereira-Sampaio M, Sampaio FJ, Favorito LA (2016). Two - part silicone mold. A new tool for flexible ureteroscopy surgical training. Int Braz J Urol.

[6] Matsumoto ED, Hamstra SJ, Radomski SB, Cusimano MD (2002). The effect of bench model fidelity on endourological skills: a randomized controlled study. J Urol.

